# Randomized Controlled Pilot Study of Transcutaneous Electrical Nerve Stimulation for Acute Back Pain in Emergency Department Patients

**DOI:** 10.5811/westjem.48818

**Published:** 2026-04-08

**Authors:** Maxwell Moor-Smith, Nicholas Kozak, Michael McCue, Julia Wilson, Tristan Jones, Samuel Brophy

**Affiliations:** * University of British Columbia, Vancouver, BC, Canada; † Island Health, Emergency Medicine, Victoria, BC, Canada; ‡ Island Health, Emergency Medicine, Port Alberni, BC, Canada; § Island Health, Pain Program, Victoria, BC, Canada

## Abstract

**Background:**

Musculoskeletal back pain is a common presenting complaint to emergency departments (ED) worldwide. In this study we aimed to evaluate the effectiveness of transcutaneous electrical nerve stimulation (TENS) as an adjunct to standard care in reducing pain for patients presenting with acute low back pain.

**Methods:**

This study has a dual-center, open-label, cluster-randomized controlled trial design. Participants were recruited from two tertiary-care EDs in Canada. We included patients with acute or acute-on-chronic back pain of < 3 weeks duration. Participants were randomized to receive either TENS for 30 minutes plus standard care, or standard care alone. We measured pain scores using the Visual Analogue Scale (VAS) at baseline, 30 minutes, and 60 minutes after initiation of the intervention. The primary outcome was the difference in mean VAS pain scores at 60 minutes between the two groups.

**Results:**

Of 94 patients considered, we enrolled 25 participants (15 control and 10 intervention). The group receiving TENS plus standard care showed a statistically significant reduction in pain scores compared to the standard care alone group at both the 30-minute (relative mean difference: 22.6%; absolute difference: 1.7 points on 10-point VAS (95% CI, −31.9%, −13.4%, P < .001) and 60-minute timepoints (relative mean difference 18.2%, absolute difference 1.4 points (−32.7%, −3.8%, P = .04). There were two return visits in the intervention group within two weeks from the index visit, and two patients reported slight discomfort with using TENS, although they kept the device on for the duration of the trial period.

**Conclusion:**

The addition of transcutaneous electrical nerve stimulation to standard care resulted in a modest but statistically significant reduction in pain scores for patients with acute back pain in the ED setting, although it did not meet our predefined threshold of clinical significance. Further research with larger sample sizes is required to clarify the effect size and role of TENS for acute back pain in the ED waiting room.

## INTRODUCTION

Back pain is one of the most common presenting complaints in the emergency department (ED), accounting for approximately 3% of all ED visits.^1^,^2^ Most of these patients will leave the ED with a non-specific diagnosis, such as mechanical low back pain, and will recover within 4–6 weeks.^3^ Pharmacologic therapy is generally limited to non-steroidal anti-inflammatory drugs (NSAID), acetaminophen, and opioids in severe cases. A recent Cochrane review found all these therapies either lack high-quality evidence for pain reduction compared to placebo or have high-quality evidence showing equivalence with placebo.^4^

Transcutaneous electrical nerve stimulation (TENS) is a non-pharmacological option for the treatment of pain based on the gate control theory: Stimulation of large myelinated fibers reduces transmission of pain through smaller nociceptive C-fibers through inhibitory actions of interneurons.^5^ The procedure is safe and inexpensive, with very few reported adverse effects and a short list of contraindications.^6^–^8^ Because professional bodies have established evidence-based safety guidelines for TENS, procedures for patient safety, consent, and monitoring are already in widespread use in physiotherapy.^9^

In 2005 a randomized controlled trial looked at pain reduction with TENS for back pain in the prehospital setting, and found a significant reduction in pain scores after 30 minutes of TENS treatment during transport of those with acute back pain lasting < 6 hours.^8^ The study was included in a 2019 systemic review from Binny et al, who found insufficient evidence to support or dismiss the use of TENS for acute low back pain. However, of three trials included in this systematic review, two evaluated the use of TENS over four-week periods, which is of questionable relevance to ED pain management.^7^ Indeed, a 2015 Cochrane review of TENS for acute pain found tentative evidence for TENS reducing pain intensity, acknowledging the high risk of bias in included trials.^6^

A 2018 pilot trial of 110 patients looked at provision of TENS in the ED for pain relief, without limitations to chief complaint. Based on survey data, the authors found 83% of patients reported a functional improvement with TENS and 100% would recommend it to a family member or friend.^10^

TENS has also shown some benefit in treating acute pelvic pain in young women,^11^ post-traumatic hip pain,^12^ and low back pain during pregnancy;^13^ it remains to be seen whether TENS has a role to play for acute back pain in the ED waiting room. If effective, TENS may have a role as an inexpensive and safe, nurse-initiated intervention that could improve the care of patients presenting to the ED with acute back pain.

## METHODS

### Study Design and Setting

We completed a dual-centre, open-label, cluster-randomized controlled trial at two tertiary-care EDs in Canada. This study was reviewed by the University of British Columbia Harmonized Ethics Board and was considered no more than minimal risk and was approved. The study is reported in accordance with the Consolidated Standards of Reporting Trials 2010 checklist for reporting a randomized trial. Our trial design was pre-published on ClinicalTrials.gov (ID: NCT05601843).

**Population Health Research Capsule**

What do we already know about this issue?

*TENS can reduce acute pain in some settings, but evidence for its effectiveness for acute back pain in the emergency department remains limited and inconsistent*.

What was the research question?

*Does adding TENS to standard care reduce acute back pain more than standard care alone in ED patients?*

What was the major finding of the study?

*TENS cut VAS pain 18.2% more than control at 60 min (1.4-pt diff; 95% CI −32.7 to −3.8; p=0.04)*.

How does this improve population health?

*TENS offers a safe, low-cost option that may reduce pain and opioid use in ED patients with acute back pain, improving comfort and supporting non-pharmacologic care*.

### Selection of Participants

We enrolled patients over two weeks in May 2023. Participants were recruited in the ED waiting room by research assistants if they had a primary triage complaint of “back pain” as per the Canadian Emergency Department Information System.^14^ We included English-speaking patients > 18 years of age who reported acute or acute-on-chronic back pain of < 3 weeks duration, had a projected wait time of at least 30 minutes, and were triaged as Canadian Triage and Acuity Scale (CTAS) between 3 and 5 in the ambulatory section of the ED. Patients were excluded if they had predetermined “red flags” on history (ie, reported fever, direct trauma, bilateral radicular symptoms, incontinence or retention of urine or stool, saddle anesthesia, or intravenous drug use within 30 days), had abnormal triage vital signs, were pregnant, had a history of epilepsy or spinal cord injury, had evidence of skin breakdown at TENS pad-placement site, or had an implanted pacemaker or neurostimulation device.

### Intervention

**Figure 1:**
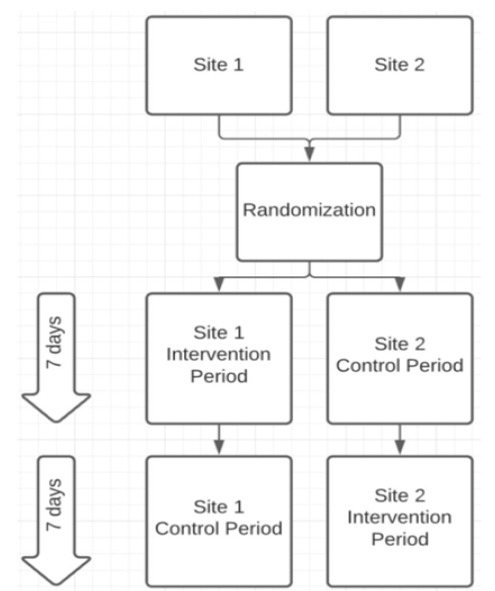
Cluster randomization by site of visit for a study of the use of transcutaneous electrical nerve stimulation for acute back pain in the emergency department.

All enrolled participants provided written informed consent, and baseline demographic data was collected. Participants were cluster randomized based on which of the two trial EDs they presented to during the trial period, with Site 1 as the control site in the first week and the intervention site for the second week, while Site 2 had the opposite schedule (see Figure 1). Participants were informed of their group assignment after enrollment. The TENS machine used for the trial was the Impulse 3000 T (2014 BioMedical Life Systems, Inc., Carlsbad, CA), applied in a frame-like pattern 3–6 cm away from the subjective area of maximal pain. The manufacturer had no role in this project or in the writing of this paper. The TENS treatment lasted 30 minutes, with the frequency set to 100 hertz and the amplitude adjusted based on participant comfort. No sham TENS was used in the control group for this trial. Standard care was provided for both the control group and TENS group, including nurse-initiated analgesia (ie, acetaminophen or ibuprofen), and any physician-ordered interventions (ie, opioids). Data on use of additional interventions (eg, trigger point injections, nerve blocks) was not collected, as this was not part of routine practice pattern at the time of this trial. The Visual Analogue Scale (VAS) was used to collect pain scores from participants after enrollment (T0), at 30 minutes (T30), and at 60 minutes (T60) after initiation of TENS treatment.

### Outcome Measures

Our primary outcome selected a priori was difference in mean VAS pain scores at T60 between the group treated with TENS + standard care, compared to standard care alone. The T60 interval was chosen as the primary endpoint to allow sufficient time for standard care (ie, pharmacotherapy) to take effect and to determine whether any benefit provided by TENS would be sustained. Difference in pain score at 30 minutes was examined as a secondary outcome. We a priori determined a 30% reduction of pain to be clinically significant based on similar thresholds reported in prior literature.^11^,^12^,^15^ We also compared the difference in opioid requirement, calculated by oral morphine equivalents (OME) used while in the ED, as well as return visits to the ED for back pain within two weeks. We did not tally non-opioid medications therapies used by participants due to lack of standard pharmacologic orders at trial sites at the time of the trial, difficulty combining data on over-the-counter use of various forms of these medications (eg, no reliable equivalency between NSAIDs or acetaminophen combination products), as well as local challenges gathering this data. Reports of adverse effects of TENS were collected and reported in a narrative format.

### Statistical Analysis

We planned to sample 20 participants (10 per study arm) over the two-week trial period based on a sample size calculation done prior to study initiation. In keeping with prior literature, a 95% power to detect an effect size of 30% at the 0.05 alpha level was used. Descriptive statistics are reported as means, standard deviations (SD), and percentages, with 95% confidence intervals for differences between groups. Comparison between groups was performed by an unpaired parametric *t*-test. We performed all analyses using Stata v17 (StataCorp, LLC, College Station, TX).

### Patient and Public Involvement

There was no involvement of patients or public in the design of this trial.

## RESULTS

94 patients were screened for participation in this trial, with 69 patients excluded. Most were excluded for not meeting inclusion/exclusion criteria (n=57, 82.6%), for which “red flags” for serious causes of back pain was the primary reason for exclusion (n=25, 36.2%). Of these, direct trauma to the back (n=11, 15.9%), bilateral radicular symptoms (n=6, 8.7%), and retention/incontinence of urine/stool (n=5, 7.2%) were the most common.

Overall, we enrolled 25 participants, of whom 52% were female with a median age of 44 (interquartile range [IQR] 34,7; 20–84), with 56% reporting a prior history of back pain. The median initial VAS pain score was 7.5 (SD 2.5; 0–10) with a mean duration of symptoms prior to presentation of 5.5 days (SD 5.2; 0.1–18). The enrollment between the sites was approximately equal, with 52% (n = 13) presenting to Site 1.

For our primary endpoint, we found a statistically significant reduction in VAS pain scores for TENS + standard care compared to standard care alone at the T60 time point, with a relative mean difference of 18.2% (95% CI, −32.7%, −3.8%, *P* = .04). This corresponds to a reduction of mean pain score of 1.5-points in the intervention group compared to a 0.1-point reduction in the control group (absolute difference of 1.4 VAS points). Similarly, there was a statistically significant reduction in pain at the T30 time point, with a mean difference of 22.6% (95% CI, −31.9%, −13.4%, *P* < .001), corresponding to a reduction of 1.8-points in the intervention group and a 0.06-point reduction in the control group (absolute difference of 1.74 VAS points). Although the reductions in VAS pain scores were statistically significant, they did not meet our a priori definition of clinical significance (a 30% reduction in pain scores). Additionally, there was a non-significant trend toward reduced opioid requirements in the control group (30.0 vs 13.8 OME, *P* = .74). (See Table 2.) Of the two patients who returned to the ED within two weeks after index visit, both were in the intervention group; one requested a note for work, and one requested further analgesia.[Fig f2-wjem-27-753][Fig f3-wjem-27-753][Table t1-wjem-27-753]

Two patients reported adverse events while using the TENS machine; one patient stated they had self-resolving light-headedness and warmth in the first five minutes, and another reported new abdominal pain. However, no patients removed the TENS machine early due to these adverse events. There were no major deviations from the protocol.[Fig f4-wjem-27-753]

## DISCUSSION

This study aimed to investigate the feasibility and effectiveness of using TENS as an adjunct to standard care for acute low back pain in the emergency department (ED). Our results demonstrate a statistically significant reduction in pain scores in the TENS group compared to the control group at both the 30-minute and 60-minute time points. While the reduction did not meet the predefined threshold for clinical significance (ie. 30%), there was a mean difference in pain reduction of 22.6% 30 minutes after TENS treatment, which was sustained at 18.2% at 60 minutes post-treatment. There was a trend toward reduced opioid use in the TENS group though this was a secondary outcome and not statistically significant. No patients removed TENS pads due to discomfort or adverse events.

Our results are consistent with prior literature demonstrating a reduction in acute pain with use of TENS. Only one prior trial has examined the effectiveness of TENS for back pain in the acute (< 3 weeks) setting, which found a statistically significant reduction in pain after a 30-minute TENS treatment during emergency transport to hospital,^8^ To our knowledge, this is the first published trial to examine the effectiveness of TENS for back pain in the ED waiting room. Several other trials have shown benefit with TENS in a variety of types of pain including renal colic, acute pelvic pain, pregnancy-related acute back pain, post-traumatic hip pain, and even unsedated colonoscopy.^6^,^11^–^13^ Taken together, these findings highlight the potential versatility and applicability of TENS in the ED setting.

The use of TENS in the ED has several advantages. It offers a safe and cost-effective alternative to pharmacological interventions, potentially reducing the reliance on opioids. Transcutaneous electrical nerve stimulation can be initiated by nurses, making it a feasible option in the ED waiting room that enhances patient care and satisfaction, particularly as average ED wait times are increasing rapidly. Furthermore, TENS can be prescribed to patients, extending the period of potential reduction of pharmacologic reliance and facilitating physical therapy.^9^

Previous research on TENS has been limited by small sample sizes, variable controls, and a lack of consistent standards for TENS dosing. Further research should focus on optimizing TENS dosing for standard study design, larger sample sizes across multiple centers, patients with historical “red flags,” and adequate blinding of participants (ie, use of a transient sham TENS device that provides < 1 minute of stimulation to reduce awareness of placebo).

## LIMITATIONS

There are several limitations for this trial. Due to funding restrictions preventing a longer period of study, this trial had a small sample size and was isolated to two urban EDs in the same city. This would limit generalizability to smaller centers or differing populations. Similarly, we had a relatively high rate of exclusions based on patient-reported “red flags” for back pain. There is no evidence that back pain with an underlying serious etiology is worsened by TENS; therefore, it may still be appropriate for these patients to receive TENS in the ED outside a research setting. Research on the use/safety of TENS in this patient subgroup should be considered.

In contrast to prior research, we elected to compare TENS with standard care against standard care alone, leaving the patients unblinded after randomization. Transcutaneous electrical nerve stimulation necessarily produces a sensation when being used, and a patient’s ability to adjust TENS amplitude would make it clear if sham TENS were being used. This approach was chosen to reflect pragmatic use of the device in the ED waiting-room environment. As pain is an inherently subjective outcome, we anticipated that by comparing the addition of TENS to standard care alone we would produce a result that more adequately informs real-world practice. Finally, our results are limited by lack of data on non-opioid and non-pharmacological analgesia received. Due to local challenges in obtaining this data, we were unable to determine whether the provision of this analgesia differed between the groups. Oral morphine equivalency use while in the ED was tallied, reflecting a surrogate measure of overall opioid requirement.

## CONCLUSION

This pragmatic, open-label, randomized controlled trial demonstrated modest effectiveness of transcutaneous electrical nerve stimulation as an adjunct to standard care for acute back pain in the ED, although this did not meet our predetermined threshold of clinical significance.

## Figures and Tables

**Figure 2 f2-wjem-27-753:**
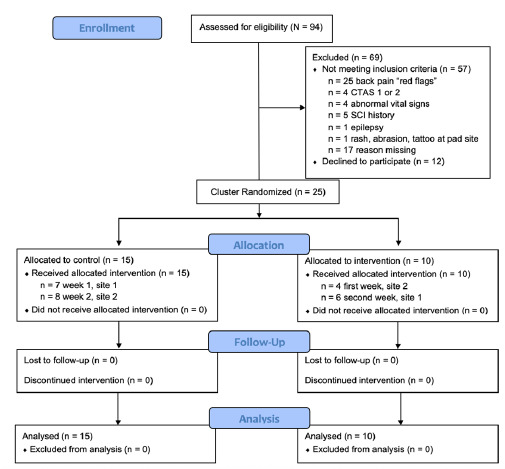
Enrollment of participants for a study examining effect of transcutaneous electrical nerve stimulation for acute back pain in the emergency department. CONSORT 2010 flow diagram. *CONSORT*, Consolidated Standards of Reporting Trials.

**Figure 3 f3-wjem-27-753:**
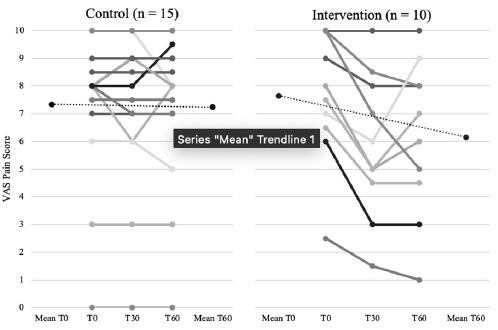
In a study of transcutaneous electrical nerve stimulation (TENS) for acute back pain in the emergency department, there was a statistically significant reduction in individual and mean Visual Analogue Scale pain scores in the intervention group (TENS + standard care) compared to the control group (standard care).

**Figure 4 f4-wjem-27-753:**
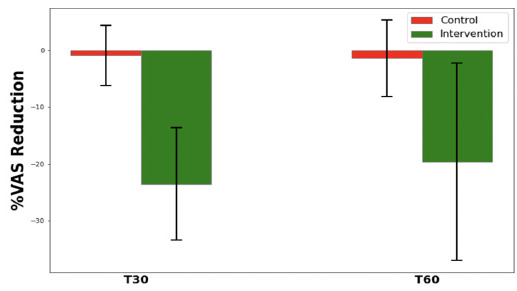
Visualization of the mean percentage reduction in VAS* pain score, at 30 and 60 minutes, between the control group and the intervention group in a study of transcutaneous electrical nerve stimulation for acute back pain in the emergency department. *VAS, Visual Analogue Scale.

**Table 1 t1-wjem-27-753:** Baseline characteristics of participants in a study of the use of transcutaneous electrical nerve stimulation for acute back pain in the emergency department.

Characteristic	Overall (N = 25)	Control (n = 15)	Intervention (n = 10)
Female sex (%)	13 (52)	6 (40)	7 (70)
Age, y, mean (SD)	49.7 (20.5)	51.5 (21.0)	47 (20.5)
Site 1 (%)	13 (52)	7 (47)	6 (60)
Previous history of back pain (%)	14 (56)	10 (67)	4 (40)
T0 VAS, mean (SD)	7.5 (2.5)	7.3 (2.6)	7.7 (2.3)
Duration, d, mean (SD)	5.5 (5.2)	7.3 (5.5)	2.6 (3.1)
Height, cm, mean (SD)	169.9 (9.0)	169 (9.5)	171.2 (8.6)
Weight, kg, mean (SD)	75.8 (19.2)	72.9 (14.3)	80.3 (25.1)
HR, mean (SD)	81.8 (14.34)	80.1 (14.4)	84.4 (14.6)
BP, mean (SD)	135.6/79.8 (17.3/14.6)	131.7/76.4 (14.9/11.3)	141.5/85 (19.7/18)

*BP*, blood pressure; *HR*, heart rate; *SD*, standard deviation; *VAS*, Visual Analogue Scale.

**Table 2:** Comparison of opioid medications provided as part of standard care between intervention and control groups, in OME (Oral Morphine Equivalency) for TENS for acute back pain in the ED. Patients may have received non-opioid analgesia but this data was not collected. There was a non-significant trend toward reduced opioid requirements in the control group (30 vs 13 OME, P =.74).

	Drug	Cumulative dose (in OME)	Total cumulative dose (in OME)
Control (n = 3)	Hydromorphone Morphine	25 mg 5 mg	30 mg
Intervention (n = 2)	Hydromorphone Tramadol	10 mg 3 mg	13 mg

*OME*, oral morphine equivalency; *SD*, standard deviation.
